# Trends in Magnesium Intake among Hispanic Adults, the National Health and Nutrition Examination Survey (NHANES) 1999–2014

**DOI:** 10.3390/nu11122867

**Published:** 2019-11-22

**Authors:** Jialiang Liu, Yuhan Huang, Qi Dai, Kimberly G. Fulda, Shande Chen, Meng-Hua Tao

**Affiliations:** 1 Department of Biostatistics and Epidemiology, University of North Texas Health Science Center, Fort Worth, TX 76107, USA; Jialiang.Liu@live.unthsc.edu (J.L.); yuhan24@outlook.com (Y.H.); 2 Department of Medicine, Division of Epidemiology, Vanderbilt Epidemiology Center, Vanderbilt University School of Medicine, Vanderbilt-Ingram Cancer Center, Vanderbilt University Medical Center, Nashville, TN 37203, USA; qi.dai@vanderbilt.edu; 3 Department of Family Medicine and Osteopathic Manipulative Medicine; NorTex, University of North Texas Health Science Center, Fort Worth, TX 76107, USA; kimberly.fulda@unthsc.edu; 4 Graduate School of Biomedical Sciences, University of North Texas Health Science Center, Fort Worth, TX 76107, USA; Shande.Chen@unthsc.edu

**Keywords:** magnesium intake, Hispanic adults, trends, gender

## Abstract

This study aimed at examining trends in magnesium intake among U.S. Hispanic adults stratified by gender, Hispanic origins, age, and poverty income ratio (PIR) level. Data on 9304 Hispanic adults aged ≥20 years from eight National Health and Nutrition Examination Survey (NHANES) cycles (1999–2014) were included in this study. For each cycle, survey-weighted mean dietary and total magnesium intakes were estimated. The prevalence of dietary and total magnesium intake below the Recommended Dietary Allowance (RDA) was further estimated stratified by gender and age groups. Linear regression was used to test trend. Over the survey cycles, both dietary and total magnesium intakes were significantly increased among Hispanic adults. In the study period, magnesium intake tended to be lower in females, adults in other Hispanic-origin group, those aged ≥65 years old, and those with a PIR <1.0. The prevalence of magnesium intake inadequacy decreased among Hispanic adults; however, more than 70% of Hispanic males and females continued to have magnesium intake below the RDA in 2013–2014. From 1999/2000 to 2013/2014, despite several improvements in magnesium intake having been identified, additional findings showed insufficient intake in Hispanic males and females, suggesting the need to improve magnesium intake through diet and dietary supplementation for U.S. Hispanics.

## 1. Introduction

Magnesium, an essential mineral, plays an important role in hundreds of physiologic activities, including energy production, lipid and glucose metabolism, and inflammation [1,2]. Magnesium is also involved in bone metabolism and the maintenance of physiological functions of bone and muscle [3,4]. Approximately 60% of total magnesium in the body is stored in bone, suggesting the importance of magnesium to the skeletal system [5]. Natural sources of magnesium include leafy green vegetables, whole grain, nuts, and milk products [6,7]. Inadequate dietary magnesium intake has been linked to various adverse health outcomes [2,6], including metabolic syndrome [8,9,10], type 2 diabetes [11,12], cardiovascular disease (CVD) [13,14], osteoporosis [15], and possibly some cancers [16,17].

The Recommended Dietary Allowance (RDA) for magnesium from all sources represents the average daily intake level that meets sufficient requirement for nearly all (97% to 98%) healthy individuals in a particular gender and life stage group [6]. For adults in the United States (U.S.), the RDA for magnesium is 400 mg/day for males aged 19–30, 420 mg/day for males ages 30 and over, 310 mg/day for females aged 19–30, and 320 mg/day for females ages 30 and over [6]. Previous surveys have shown that magnesium intake has been historically low in the U.S. population [1,18]. The 2001–2008 National Health and Nutrition Examination Survey (NHANES) showed that over 50% of the adult population did not consume the recommended magnesium intake regardless of their weight status [18]. The 2015–2020 Dietary Guideline for Americans particularly identified magnesium as one of the shortfall nutrients [19].

In the U.S., the Hispanic population constitutes the youngest and rapidly growing racial/ethnic group [20,21]. The Hispanic population is estimated to increase to 132.8 million (30% of the U.S. population) in 2050, with the largest increase in Mexican Americans [22]. Moreover, Hispanic adults have a higher prevalence of metabolic syndrome [23] and diabetes mellitus [24] than non-Hispanic whites. However, current knowledge about the status of magnesium intake in this population remains very limited. There is a critical need to understand trends in magnesium intake and corresponding disparities in the U.S. Hispanic population, which may contribute to inform priorities to improve diets, promote healthy diet behavior, as well as to prevent associated health consequences.

In the present study, data from the NHANES 1999–2014 were used to examine trends in dietary and total magnesium intake among U.S. Hispanic adults stratified by gender, ethnicity origins, age, and poverty income ratio (PIR) level. Additionally, the temporal trends in the prevalence of total magnesium intake below the RDA were evaluated among Hispanic adults by gender and age group.

## 2. Materials and Methods

### 2.1. Study Population

Data from eight continuous cycles of the National Health and Nutrition Examination Survey (NHANES) between 1999 and 2014 were used in this study. The NHANES is a cross-sectional survey designed to monitor the health and nutritional status of a nationally representative sample of the noninstitutionalized civilian U.S. population [25]. The NHANES data are released every two years by the U.S. National Center for Health Statistics (NCHS) of the Centers for Disease Control and Prevention [25]. Our study population included adults aged ≥20 years at the time of the survey, who self-identified as Mexican Americans or other Hispanic race/ethnicity, and with at least 1 valid 24 h dietary recall determined by the NCHS (*n* = 10,423). Pregnant or lactating females and respondents who had missing data for magnesium intake were excluded from the analysis. Finally, a total of 9304 participants were included in this analysis. The sample size per cycle ranged from 911 to 1603. All participants provided written informed consent, and the Research Ethics Review Board at the (NCHS) approved the survey protocol [25].

### 2.2. Assessments of Magnesium Intake

Details of the protocol and dietary data collection methods are fully described elsewhere [26]. Briefly, daily dietary magnesium intake information was obtained through 24 h recall interviews using the U.S. Department of Agriculture’s Automated Multiple-Pass method [27,28]. Prior to 2003, the NHANES only collected dietary intake with one 24 h dietary recall for all participants. From 2003, the NHANES collected two 24 h recalls for each participant. The first dietary recall was collected in person by trained interviewers in NHANES mobile examination centers (MEC). The second dietary recall was completed by telephone 3–10 days after the MEC interview [29]. Dietary supplement information was collected during the household interview, and since 2007, was collected in two 24 h recalls, as a part of the Dietary Supplement Questionnaire [26]. Information included the participant’s use of vitamins, minerals, and other dietary supplements over the past 30 days [26]. Moreover, information about type, consumption frequency, duration, and amount taken was also collected for each reported dietary supplement [26]. The average daily magnesium intake from dietary supplements was calculated for participants using the number of days supplement use was reported, the reported amount taken per day, and the serving size unit from the product label [28]. Total magnesium intake was calculated by summing intakes from diet and supplements.

### 2.3. Statistical Analysis

All statistical analyses were conducted in SAS software (version 9.4, SAS Institute, Cary, NC, USA) using the “Survey” procedures to incorporate the complex, multistage survey sampling design of the NHANES. Survey-weighted means and standard errors (SE) for dietary and total magnesium intakes were estimated for each NHANES cycle among all Hispanic adults, as well as subpopulations stratified by age (i.e., 20–34, 35–49, 50–64, and ≥65 years), gender, ethnicity origins (i.e., Mexican American, other Hispanic), and PIR level (i.e., <1.0, 1.0–1.84, and ≥1.85). The prevalence of magnesium intake below the RDA was estimated stratified by gender and age groups for each NHANES cycle between 2003 and 2014 which had two nonconsecutive 24 h recalls using the established National Cancer Institute (NCI) method [30]. Complete details of the NCI method are shown elsewhere, and the SAS macros necessary to estimate the distribution of a nutrient intake below a certain threshold are available on the NCI website [30]. Time trends in the mean dietary, total magnesium intakes, and prevalence of magnesium intake inadequacy were examined by survey-weighted linear regression models. The statistical significance of trends was assessed by treating survey year as a continuous independent variable. The trends were tested in the overall population, as well as in each subpopulation stratified by gender, ethnicity origins, age and PIR categories. All statistical tests were based on two-sided probability and a significance level of *p* < 0.05.

## 3. Results

Over the survey cycles, the proportion of Hispanic adults aged 35–49 years old increased, while the proportion of Hispanic adults aged 50 years and above decreased (Table 1). The percentage of Hispanic males slightly increased over the study period. The proportion of Mexican American adults increased from 46.3% in 1999–2000 to 61.1% in 2013–2014, and other Hispanic adults decreased. The proportion of Hispanic adults with PIR < 1.0 increased from 25.4% in 1999–2000 to 31.1% in 2013–2014.

Trends in mean dietary and total intakes of magnesium among Hispanic adults ≥20 years old and subpopulations from 1999 to 2014 are presented in Table 2 and Table 3, respectively. For the daily dietary magnesium intake, the average intake significantly increased from 275.06 mg in 1999–2000 to 319.21 mg in 2013–2014, with an improvement of 16.1% in overall Hispanic adults aged ≥20 years old (*p*-trend < 0.001) (Table 2). In overall Hispanic adults, the average dietary magnesium intake increased between 1999–2000 and 2005–2006, slightly decreased between 2005–2006 and 2007–2008, thereafter increased and peaked in 2013–2014. Thus, there were significant trends in the mean dietary magnesium intake in all subgroups (all *p*-trends < 0.001). Significant increases in dietary magnesium intake were further observed among both genders, all ages, both Hispanic-origin subgroups, and all PIR levels.

For the trend in total magnesium intake, a similar pattern with the trend in dietary magnesium intake was found across the survey cycles among overall Hispanic adults ≥20 years old (Table 3). The mean intake increased from 303.89 mg/day in 1999–2000 to 331.78 mg/day in 2013–2014, with an improvement of 9.2% among all Hispanic adults ≥20 years old (*p*-trend < 0.001). Furthermore, the mean total magnesium intake significantly increased among Hispanic adults in both genders, all age subgroups, and both Hispanic-origin subgroups (*p*-trends < 0.001). By PIR levels, total magnesium intakes were significantly increased among Hispanic adults, with PIR lower than 1.85 across survey cycles, while a significant reduction in total magnesium intake was observed among Hispanic adults with PIR ≥ 1.85 between 1999–2000 and 2013–2014 (*p*-trend < 0.001). In all survey cycles, females, other Hispanics, adults aged 65 years and above, and adults with PIR < 1.0 tended to have lower dietary and total magnesium intakes.

**Figure 1:**
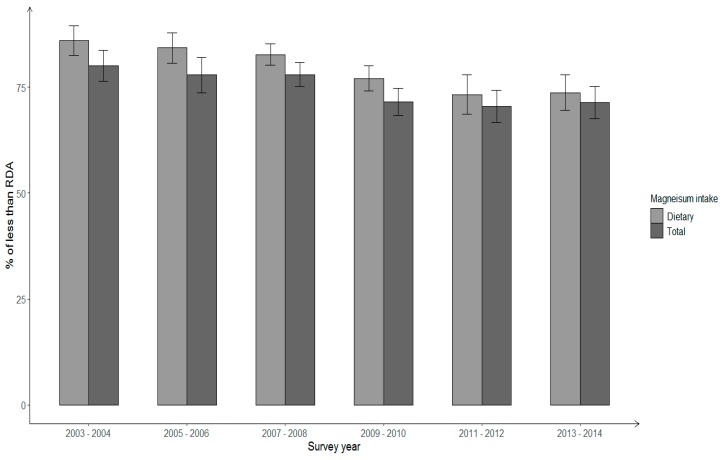
Percentage of insufficient dietary and total magnesium intake among Hispanic males aged ≥20 years across NHANES survey years 2003–2014.

**Figure 2:**
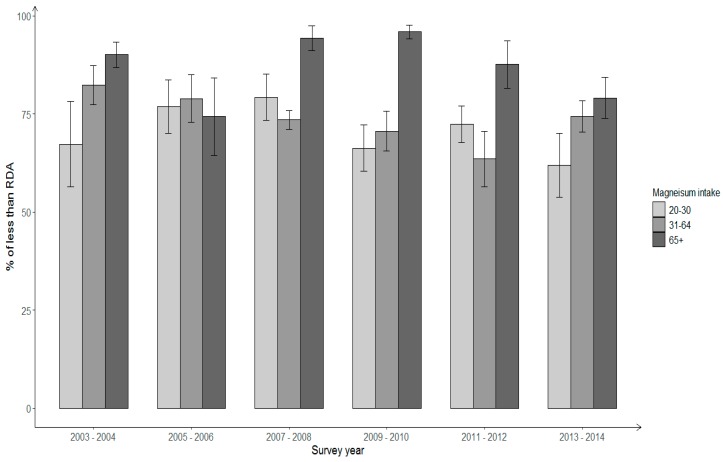
Percentage of insufficient total magnesium intake among Hispanic males by age groups across NHANES survey years 2003–2014.

Trends in the proportion of Hispanic adults with dietary and total magnesium intake not meeting the RDA were further evaluated by gender and age groups. Significant decreases were observed for the estimated prevalence of Hispanic males ≥20 years old consuming dietary and total magnesium intakes less than the RDA (*p* for linear trend < 0.05) (Figure 1). Between 2003–2004 and 2013–2014, the prevalence of inadequate magnesium intake from foods decreased from 86% to 73.7%, and the prevalence of total magnesium intake less than the RDA reduced from 80.1% to 71.4%. By age groups, improvement of total magnesium intake was modest among Hispanic males aged 31–64 years (*p* for linear trend = 0.05), with the prevalence of total magnesium intake below the RDA decreasing from 82.3% to 74.3% between 2003–2004 and 2013–2014 (Figure 2). Although the prevalence of total magnesium intake less than the RDA also decreased among Hispanic males in 20–30-year and ≥65-year groups, the changes were not statistically significant.

**Figure 3:**
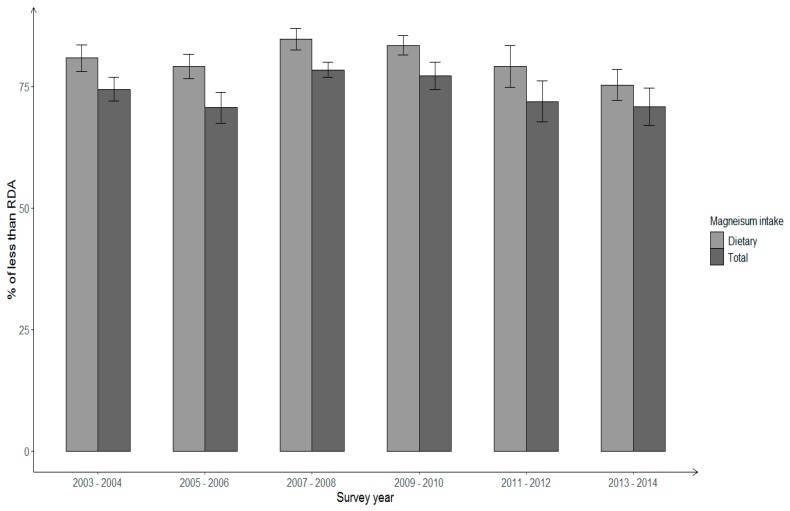
Percentage of insufficient dietary and total magnesium intake among Hispanic females aged ≥20 years across NHANES survey years 2003–2014.

Among overall Hispanic females ≥20 years, the prevalence of magnesium intake below the RDA decreased from 80.8% in 2003–2004 to 75.3% in 2013–2014 for dietary intake, and the corresponding change for total magnesium intake was 74.4% to 70.8%; however, the changes were not statistically significant (*p* for linear trend > 0.05) (Figure 3). In addition, there was no significant trend in the prevalence of Hispanic females not meeting the RDA for total magnesium intake in all age groups (figure not shown). The prevalence with total magnesium intake less than the RDA decreased from 76.1% in 2003–2004 to 66.6% in 2013–2014 among Hispanic females aged 31–64 years; whereas, it increased among females 20–30-year-old (from 73.9% to 76.7%) and females aged ≥65 years old (from 59.6% to 69.5%), reflecting differences by age. In addition, the prevalence of insufficient total magnesium intake in different age groups varied by gender. Over time, among Hispanic adults less than 65 years, the proportion of males with total magnesium intake not meeting the RDA tended to be less than the prevalence among females. However, in the senior group (≥65 years) the prevalence was higher among Hispanic males than among females. In 2013–2014, more than 70% of Hispanic adults still had a total magnesium intake below the RDA regardless of their age and gender.

## 4. Discussion

Based on nationally representative data collected between 1999–2000 and 2013–2014, magnesium intake improved significantly among U.S. Hispanic adults, with 16.1% improvement for the mean dietary magnesium intake and 9.2% improvement for the mean total magnesium intake. Despite observed overall improvements, trends in magnesium intake from all sources varied by PIR levels, with a trend of reduction among Hispanic adults with a higher PIR. Meanwhile, there were persistent comparative differences in dietary and total magnesium intakes by gender, age and ethnicity subgroups, and PIR levels, with lower intakes among Hispanic females, other Hispanics, adults aged ≥65 years, and adults with lower PIR over the survey cycles. Furthermore, although magnesium intake increased over the study period, 71.4% and 70.8% of Hispanic males and females had total magnesium intake less than the RDA in 2013–2014, respectively.

Between 1999–2000 and 2013–2014, total magnesium intake increased significantly among Hispanic adults with PIR < 1.85 but declined among those with PIR ≥ 1.85. One possible explanation for this difference in trend may be that Hispanic adults with high family income are more likely to adopt the standard American diet, which is generally characterized by a high intake of fat and sugar, and low consumption of nutrient-dense food [31]. A previous study found that Hispanic women with a higher education attainment were more likely to have a higher intake of fat [32]. Because PIR level is positively correlated with education attainment, Hispanic adults with a higher PIR level may be more likely to be exposed to typical American diets. These findings suggest that socioeconomic factors such as education and family income may be an important proxy of acculturation. 

Between 1999–2000 and 2013–2014, magnesium intake increased significantly among Hispanic males and females, while Hispanic females persistently had lower intakes than Hispanic males. The reason for the gender difference in magnesium intake is unknown. Possible explanations for the gender differences include different biological requirements for men and women to maintain magnesium homeostasis in the body [6], and differences in body size, and physical activities between men and women [33], which could lead to gender differences in magnesium intake. 

Magnesium is widely distributed in plant and animal foods [6,7]; however, a large proportion of U.S. adults do not consume a sufficient amount of magnesium as they are recommended [19]. Dietary supplementation is an important source of magnesium, and the most commonly used adult multivitamin/minerals contain 50 mg of magnesium per serving [34]. In further analysis, we found that the prevalence of using magnesium supplements remained low among Hispanic adults, ranging from 18.7% to 25.5% across survey years. Hispanic adults who used magnesium supplements consistently reported higher dietary magnesium intake across survey cycles compared to those who did not use a magnesium supplement (data not shown). In 2013–2014, the last study survey cycle, the average daily intakes of dietary magnesium were 405.46 mg for Hispanic male supplement users and 276.17 mg for female supplement users, but the average intakes of dietary magnesium were only 357.79 mg/day and 271.82 mg/day for Hispanic male and female supplement non-users, respectively. Previous studies have reported that calcium supplement users were more likely to have a higher dietary intake of calcium than those who do not use supplemental calcium [35]. These findings suggest that individuals who consume dietary supplements (i.e., magnesium or calcium) may have healthier dietary habits. The high prevalence of supplement non-users in Hispanic adults over the survey cycles may partially explain why the prevalence of magnesium intake below the RDA remained high in 2013–2014.

One strength of our study is the use of data from the NHANES with a nationally representative sample of the U.S. population, and oversampled Hispanic subjects, which provided a sufficient sample of Hispanic adults from different demographic subgroups to provide sufficient statistical power. Several limitations should also be mentioned. Self-reported supplement intake is subject to recall bias; however, participants were asked to provide their supplement bottles during the in-person interviewing. Although multiple 24 h dietary recalls are used as a gold standard measure in nutritional epidemiological studies [30], a one-time 24 h dietary recall may not capture long-term magnesium intake. Self-reported dietary recall may result in both random and systematic errors with the potential for recall bias to occur [36].

In conclusion, our results showed improvements in both dietary and total magnesium intakes among U.S. Hispanic adults between 1999 and 2014; however, magnesium consumption remains suboptimal among the U.S. Hispanic population. Insufficient magnesium intake has been associated with a higher risk of type 2 diabetes [11,12] and other chronic diseases [13,17], and there is an increase in the rate of type 2 diabetes in the U.S. Hispanic population [20]. Findings from this study may inform discussions on emerging successes, areas for more attention, and opportunities to develop appropriate, multi-level prevention approaches to improve magnesium intakes from diet and dietary supplementation for Hispanic adults living in the United States.

## Figures and Tables

**Table 1:** Demographics of Hispanic adults aged ≥20 years by National Health and Nutrition Examination Survey (NHANES) cycle, 1999–2014 (*n* = 9304).

Demographics ^a^	1999–2000	2001–2002	2003–2004	2005–2006	2007–2008	2009–2010	2011–2012	2013–2014
	(*n* = 1288)	(*n* = 1087)	(*n* = 928)	(*n* = 911)	(*n* = 1475)	(*n* = 1603)	(*n* = 914)	(*n* = 1098)
Age, years								
20–34	314 (39.0)	343 (41.1)	231 (39.4)	324 (42.1)	396 (38.5)	426 (37.7)	252 (39.2)	277 (37.2)
35–49	345 (31.9)	335 (34.8)	244 (37.0)	271 (34.7)	390 (33.9)	477 (36.4)	240 (32.6)	334 (35.9)
50–64	318 (18.9)	209 (14.8)	183 (14.6)	183 (15.6)	431 (18.8)	440 (16.9)	273 (19.7)	300 (18.3)
≥65	311 (10.2)	200 (9.3)	270 (8.9)	133 (7.6)	258 (8.8)	260 (9.1)	149 (8.4)	187 (8.6)
Gender								
Male	633 (49.2)	574 (53.0)	488 (53.8)	491 (54.1)	711 (53.8)	779 (53.4)	471 (52.3)	528 (51.4)
Female	655 (50.8)	513 (47.0)	440 (46.2)	420 (45.9)	764 (46.2)	824 (46.6)	443 (47.7)	570 (48.6)
Race/Ethnicity								
Mexican American	1037 (46.3)	902 (52.8)	808 (68.1)	784 (68.8)	894 (63.1)	1029 (62.8)	456 (55.1)	652 (61.1)
Other Hispanic	251 (53.7)	185 (47.2)	120 (31.9)	127 (31.2)	581 (36.9)	574 (37.2)	458 (44.9)	446 (38.9)
PIR ^b^								
<1.0	307 (25.4)	256 (28.2)	267 (27.4)	266 (27.5)	364 (28.5)	436 (33.3)	264 (32.1)	297 (30.1)
1.0–1.84	374 (32.7)	301 (28.1)	252 (25.4)	239 (29.4)	376 (29.4)	387 (28.0)	222 (25.3)	256 (26.8)
≥1.85	398 (41.9)	450 (43.7)	359 (47.2)	347 (43.2)	553 (42.1)	537 (38.7)	344 (42.6)	413 (43.2)
Supplement use								
Yes	291 (25.5)	255 (22.1)	244 (23.9)	206 (23.8)	335 (20.6)	367 (21.9)	187 (19.7)	226 (18.7)

^a^ Values are presented as frequency (weighted percentages); ^b^ the numbers may not sum to the total number of participants due to missing data. PIR: poverty income ratio.

**Table 2:** Trends in daily dietary magnesium intake (mg/day) among Hispanic adults aged ≥20 years, the National Health and Nutrition Examination Survey (NHANES) 1999–2014* (*n* = 9304).

Factors	1999–2000	2001–2002	2003–2004	2005–2006	2007–2008	2009–2010	2011–2012	2013–2014	*p*-Value for Trend ^a^
	(*n* = 1288)	(*n* = 1087)	(*n* = 928)	(*n* = 911)	(*n* = 1475)	(*n* = 1603)	(*n* = 914)	(*n* = 1098)	
Overall	275.06 ± 7.20	275.72 ± 7.49	290.87 ± 6.15	302.80 ± 7.21	296.40 ± 7.45	307.19 ± 5.35	315.06 ± 8.69	319.21 ± 7.54	<0.0001
Gender									
Male	317.08 ± 10.31	317.92 ± 6.91	328.15 ± 10.28	333.57 ± 9.31	336.31 ± 7.88	354.59 ± 8.33	359.79 ± 11.90	364.68 ± 9.79	<0.0001
Female	236.62 ± 11.98	227.57 ± 9.69	250.07 ± 7.44	265.70 ± 7.18	250.86 ± 8.38	255.87 ± 6.39	266.02 ± 7.63	272.82 ± 6.86	<0.0001
Race/Ethnicity									
Mexican American	282.49 ± 8.39	292.79 ± 5.98	299.91 ± 7.61	311.02 ± 9.29	299.20 ± 9.31	320.87 ± 7.02	327.79 ± 11.96	327.83 ± 10.79	<0.0001
Other Hispanic	269.09 ± 9.93	254.14 ± 8.50	267.99 ± 13.01	279.56 ± 14.18	291.65 ± 9.43	284.98 ± 6.86	299.05 ± 10.40	305.20 ± 10.29	<0.0001
Age, years									
20–34	284.31 ± 11.01	278.37 ± 8.40	319.75 ± 12.95	301.16 ± 8.93	293.57 ± 12.46	316.10 ± 9.78	320.33 ± 11.77	324.83 ± 13.60	<0.0001
35–49	283.87 ± 10.34	278.09 ± 8.34	278.93 ± 12.54	317.90 ± 9.35	318.85 ± 10.85	318.74 ± 10.03	329.81 ± 11.60	319.28 ± 4.37	<0.0001
50–64	264.81 ± 18.80	290.35 ± 16.76	264.00 ± 13.11	299.99 ± 20.03	290.48 ± 7.83	290.20 ± 5.95	304.61 ± 12.25	326.90 ± 12.14	<0.0001
≥65	231.40 ± 18.15	231.57 ± 12.79	255.64 ± 13.90	259.46 ± 18.71	241.18 ± 6.21	258.69 ± 7.797	264.52 ± 10.47	280.61 ± 10.67	<0.0001
PIR									
<1.0	240.83 ± 11.90	240.97 ± 15.78	261.40 ± 12.47	290.48 ± 17.98	277.88 ± 16.53	300.17 ± 8.08	302.61 ± 14.66	298.43 ± 9.15	<0.0001
1.0–1.84	263.40 ± 13.96	273.43 ± 11.46	305.30 ± 12.46	298.77 ± 12.12	283.43 ± 12.84	309.75 ± 7.59	320.73 ± 17.25	346.06 ± 13.36	<0.0001
≥1.85	303.08 ± 13.07	297.16 ± 8.55	299.52 ± 10.25	308.57 ± 11.91	316.34 ± 6.28	310.82 ± 10.69	320.48 ± 9.47	318.31 ± 9.44	<0.0001

* Values are presented as weighted means ± standard error. ^a^ *p* value from linear regression by modeling survey period as a continuous variable.

**Table 3:** Trends in total magnesium intake (mg/day) among Hispanic adults aged ≥ 20 years, the National Health and Nutrition Examination Survey (NHANES) 1999–2014* (*n* = 9304).

Factors	1999–2000	2001–2002	2003–2004	2005–2006	2007–2008	2009–2010	2011–2012	2013–2014	*p*-Value for Trend ^a^
	(*n* = 1288)	(*n* = 1087)	(*n* = 928)	(*n* = 911)	(*n* = 1475)	(*n* = 1603)	(*n* = 914)	(*n* = 1098)	
Overall	303.89 ± 7.35	296.74 ± 6.73	312.43 ± 5.95	323.68 ± 8.36	313.97 ± 8.27	323.28 ± 5.78	329.90 ± 9.18	331.78 ± 8.11	<0.0001
Gender									
Male	343.22 ± 8.06	340.40 ± 6.67	347.55 ± 11.47	353.72 ± 10.69	356.22 ± 10.95	370.19 ± 8.65	372.67 ± 12.35	373.03 ± 10.47	<0.0001
Female	267.92 ± 14.48	246.92 ± 9.48	273.99 ± 6.32	287.47 ± 7.92	265.77 ± 7.56	272.49 ± 8.23	283.00 ± 7.77	289.70 ± 8.37	<0.0001
Race/Ethnicity									
Mexican American	307.88 ± 8.78	316.66 ± 6.72	320.00 ± 8.29	331.09 ± 10.62	312.56 ± 10.21	338.18 ± 7.18	343.74 ± 12.75	338.67 ± 11.30	<0.0001
Other Hispanic	300.69 ± 9.82	271.56 ± 7.78	293.26 ± 12.98	302.75 ± 15.14	316.34 ± 13.46	299.08 ± 7.86	312.48 ± 11.03	320.59 ± 10.51	<0.0001
Age, years									
20–34	302.85 ± 10.52	292.77 ± 6.24	329.04 ± 12.58	315.91 ± 11.11	311.09 ± 14.78	328.27 ± 9.71	325.15 ± 11.89	330.46 ± 13.58	<0.0001
35 – 49	318.30 ± 12.99	299.24 ± 9.86	297.57 ± 11.63	340.48 ± 12.40	333.25 ± 10.27	332.38 ± 11.35	353.80 ± 14.62	330.91 ± 5.85	<0.0001
50 – 64	305.08 ± 25.58	322.17 ± 15.66	306.78 ± 20.91	320.92 ± 15.85	312.61 ± 8.53	316.09 ± 7.75	324.81 ± 14.65	341.32 ± 13.67	0.0010
≥ 65	260.98 ± 12.22	264.64 ± 15.28	305.24 ± 22.85	301.27 ± 22.14	260.67 ± 7.91	281.71 ± 7.17	278.84 ± 12.93	320.26 ± 21.53	0.0046
PIR									
<1.0	263.48 ± 11.03	265.28 ± 15.79	271.48 ± 13.14	297.22 ± 18.19	286.04 ± 16.83	311.95 ± 8.36	310.76 ± 14.86	308.53 ± 9.74	<0.0001
1.0–1.84	283.82 ± 13.83	287.52 ± 9.48	330.10 ± 11.56	320.32 ± 9.39	299.26 ± 13.56	323.62 ± 8.21	328.20 ± 17.03	361.14 ± 12.52	<0.0001
≥1.85	341.34 ± 13.44	323.35 ± 9.67	326.78 ± 10.91	338.44 ± 13.24	343.16 ± 9.64	333.50 ± 12.57	346.33 ± 10.35	331.97 ± 10.37	<0.0001

* Values are presented as weighted means ± standard error. ^a^ *p* value from linear regression by modeling survey period as a continuous variable.
